# The functional structure of foxtail millet rhizoplane microbiome and its association with yield

**DOI:** 10.1128/spectrum.00707-26

**Published:** 2026-07-17

**Authors:** CanZhi Jin, Qiuge Chen, Xin Liu, Huan Liu, Yayu Wang

**Affiliations:** 1 College of Life Sciences, University of Chinese Academy of Sciences 74519, Beijing, China; 2 State Key Laboratory of Genome and Multi-omics Technologies, BGI Research 672740, Shenzhen, China; 3 School of Agriculture, Yunnan University 12635, Kunming, China; Instituto de Ecología, A.C. (INECOL), Pátzcuaro, Michoacán, Mexico

**Keywords:** millet, rhizoplane microbiomes, network, metagenome

## Abstract

Root-associated microbial communities profoundly influence plant growth and productivity. Although the rhizosphere microbiome has been extensively studied, the functional distinctiveness and host-specific role of the closely adhering rhizoplane microbiota remain unclear. In this study, we performed deep metagenomic sequencing of both the rhizosphere and rhizoplane microbiomes in foxtail millet (*Setaria italica*). We constructed a comprehensive non-redundant gene catalog, reconstructed 595 metagenome-assembled genomes (MAGs), and analyzed the co-occurrence networks. Our results revealed that the rhizoplane sustains a core microbial network with greater complexity and connectivity than rhizospheres. Metabolically, the rhizoplane microbiome is enriched in the functions underlying host adaptation, including ammonium production and polysaccharide decomposition. Our results showed that the associations between microbial features (taxonomic and functional) and yield were significantly stronger in the rhizoplane than in the rhizosphere. We identified 22 yield-positive MAGs, primarily from Bacillales, harboring genes for plant growth-promoting traits, such as nutrient solubilization and phytohormone synthesis. Collectively, our findings illustrate that the rhizoplane is not only a subset of the rhizosphere but also a critical host-microbe interface and functional hotspot where specialized microbial processes are directly coordinated to enhance plant performance and yield.

**IMPORTANCE:**

Plant roots selectively recruit diverse and beneficial microorganisms from the surrounding soil, assembling a distinctive rhizosphere microbiome. Substantial research, primarily utilizing amplicon sequencing, has elucidated the taxonomic composition of these rhizosphere communities across a wide range of plant species. The functional architecture, assembly processes, and coexistence mechanisms of the rhizoplane microbiome remain poorly understood, and their link to host plant traits is unclear. We elucidate the taxonomic and functional structural disparities between the rhizosphere and rhizoplane microbiomes, thereby clarifying the composition and functional roles of the rhizoplane microbiome, and further examine the association between the rhizoplane microbiome and millet yield. A deeper understanding of root-associated microbial communities may inform the development of effective agricultural probiotics, thereby enhancing sustainable farming practices. Additionally, the candidate biomarkers identified in this work offer potential targets for improving cultivation practices and supporting the long-term agricultural sustainability of foxtail millet.

## INTRODUCTION

In nature, land plants establish an intimate relationship with soil-borne microbiota, which plays a pivotal role in plant growth and health in both direct and indirect ways (1–3). The rhizosphere (a narrow zone of soil near roots) and rhizoplane (root surface) assemble distinct microbial communities compared to bulk soil (4, 5). Recently, the rhizosphere microbiome has been comprehensively studied by 16S rRNA gene amplicon sequencing in diverse plant species, including *Arabidopsis thaliana*, rice, grapevine, foxtail millet, maize, and barley (4–9). A consistent trend shows that root-associated microbiomes mainly include Pseudomonadota, Actinomycetota, Bacteroidota, and Bacillota. Some bacteria with well-characterized plant growth-promoting traits such as nitrogen fixation, phosphate solubilization, siderophore, and phytohormone production are observed in these phyla (10).

As taxonomic compositions do not necessarily reflect the functional compositions of microbiomes (11), the investigation of the functional composition of root microbiomes by metagenomic sequencing is emerging. Pioneer work has described the functional compositions of rhizosphere microbiomes by metagenomic sequencing, showing that several pathways, such as flagellar assembly, quorum sensing, bacterial chemotaxis, type III and IV secretion systems (T3SS and T4SS), biofilm formation, siderophore production, and sugar transport, were enriched in the rhizosphere microbiome when compared with the corresponding bulk soil microbiome (8, 9, 12, 13). These enriched functional traits suggest a functionally intimate relationship between the rhizosphere microbiome and host plants. Although in-depth research has improved our understanding of the nature of the rhizosphere, the differences in functional composition between the rhizoplane and rhizosphere have been examined in only a limited number of studies (14) and remain relatively underexplored, warranting further investigation. Addressing this gap will help advance our understanding of the microbial assemblage process in this microecosystem.

Foxtail millet (*Setaria italica*) is an important minor crop in East Asia and has been domesticated in China for more than 8,700 years. It is highly praised for its high nutritional value, including essential amino acids, fatty acids, and minerals, and is considered to be one of the most digestible and non-allergenic grains (15). Currently, it is grown on approximately 2 million hectares and produces nearly 6 million tons of grains each year in China (16). Our previous study has revealed the root bacterial community of foxtail millet and its potential effects on host plant productivity by analyzing around 3,000 amplicon samples (5); however, understanding the functional roles these microbes play in host phenotypes is a cornerstone to further monitor soil fertilities and amend foxtail millet growth. The rhizoplane microbiome is more tightly attached to the root surface than the rhizosphere microbiome and may therefore establish a more active relationship with the plant, potentially making it highly relevant to plant phenotypes.

To enable comprehensive metagenomic exploration and to elucidate the functional architecture of the rhizosphere and rhizoplane microbiome—as well as the relationships between rhizoplane microbial communities and host plant yield—we conducted deep metagenomic sequencing of the rhizosphere and rhizoplane microbiome of foxtail millet.

## RESULTS

### Rhizoplane compartment shape root-associated microbial community structure and stability

**Fig 1:**
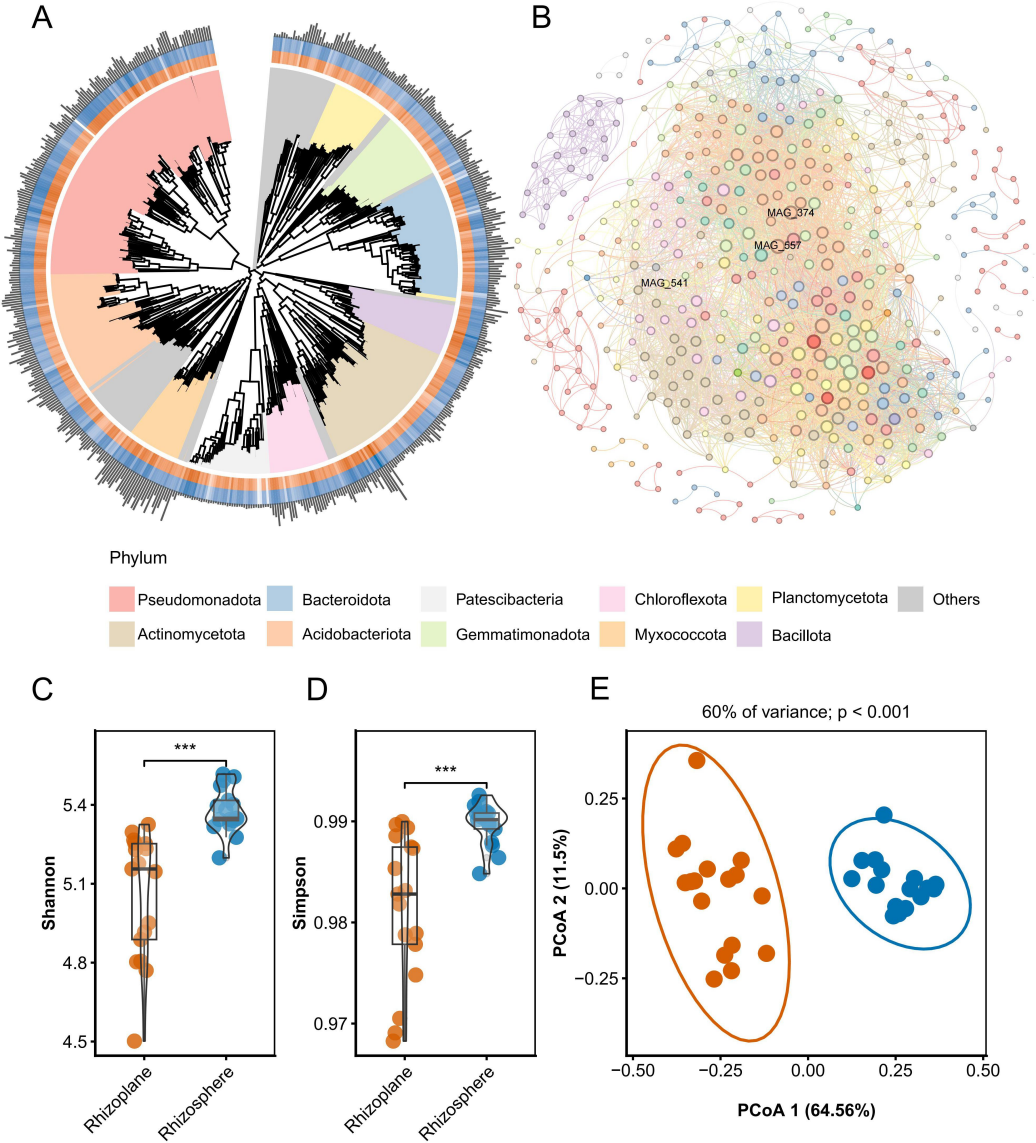
Phylogenetic tree, co-occurrence network, and diversity of metagenome-assembled genomes (MAGs). (**A**) Phylogenetic tree of 595 MAGs. MAGs are colored by phylum as shown in the legend. Inner ring: MAG abundance in rhizoplane samples (orange) and rhizosphere samples (blue); outer ring: genome size distribution (scale in Mb). (**B**) Co-occurrence network of MAGs; nodes represent individual MAGs (colored by phylum), and edges indicate correlations. Hub nodes are labeled with text. (**C** and **D**) Comparison of alpha diversity (Shannon index, **C**; Simpson index, **D**) between rhizoplane (orange) and rhizosphere (blue) niches. Significance was assessed using the Wilcoxon rank-sum test (*** indicates *P* < 0.001). Box plots show the median (center line) and the 25th and 75th percentiles (box). (**E**) Principal coordinate analysis (PCoA) plot based on Bray-Curtis dissimilarity of MAGs.

We generated over 3.06 terabases (Tb) of metagenomic data, including 34 paired, deep-sequenced rhizoplane and rhizosphere samples averaging 30.24 gigabases (Gb) of clean data per sample, and 6 ultra-deep-sequenced samples averaging 332.78 Gb of clean data per sample. Then, we reconstructed 595 non-redundant metagenome-assembled genomes (MAGs), which covered 25 bacterial phyla and 1 archaea phylum (Table S1). Pseudomonadota was the most abundant phylum, represented by 139 MAGs, followed by Actinobacteriota (72 MAGs), Bacteroidota (64 MAGs), Actinomycetota (63 MAGs), and Patescibacteria (40 MAGs). Thermoproteota was the sole archaeal phylum identified, comprising 12 MAGs (Fig. 1A). We first constructed a co-occurrence network of all samples, in which contained 437 MAGs and 5,671 edges, and identified 3 MAGs as hub nodes (MAG_374, MAG_541, and MAG_557), from Vicinamibacterales and Gemmatales orders (Fig. 1B).

**Fig 2:**
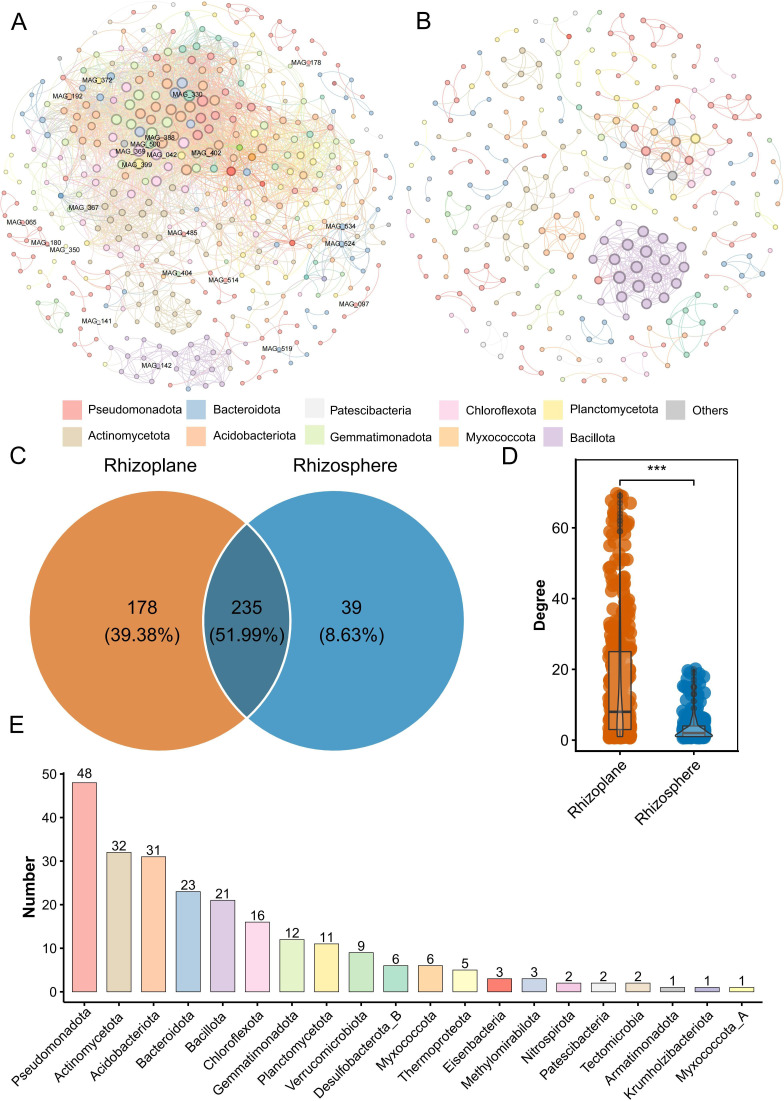
Co-occurrence networks of metagenome-assembled genomes (MAGs) in the rhizoplane and rhizosphere. (**A** and **B**) Co-occurrence networks of MAGs from the rhizoplane (**A**) and rhizosphere (**B**); nodes represent individual MAGs and are colored by phylum. Hub nodes are labeled with text. (**C**) Venn diagram showing the overlap of MAGs between the rhizoplane (orange) and rhizosphere (blue) networks, with 235 shared MAGs. (**D**) Comparison of node degrees between rhizoplane and rhizosphere networks. Significance was assessed using the Wilcoxon rank-sum test (*** indicates *P* < 0.001). (**E**) Phylum-level taxonomic distribution of the 235 MAGs shared between the rhizoplane and rhizosphere networks.

To further investigate the distinct structure of microbial communities in the rhizoplane versus the rhizosphere, we analyzed paired samples from each compartment separately. The rhizosphere microbiome exhibited significantly higher MAG diversity (pairwise Wilcoxon rank-sum test, *P*-value < 0.001) than the rhizoplane microbiome (Fig. 1C and D). Furthermore, the microbial community composition differed substantially between the two niches (permutational multivariate analysis of variance [PERMANOVA], *P* < 0.001; Fig. 1E). We then constructed separate co-occurrence networks for the rhizoplane and rhizosphere niches (Fig. 2A and B). The rhizoplane network was substantially larger and more complex, comprising 413 MAGs and 3,248 edges (Table S2). In contrast, the rhizosphere network was less densely connected, containing only 274 MAGs and 493 edges.

A total of 235 MAGs were shared between the rhizoplane and rhizosphere networks (Fig. 2C), representing a conserved core that underpins the architecture of the root-associated microbial community. Besides, MAGs in the rhizoplane network possessed significantly higher degree centrality than those in the rhizosphere network (Wilcoxon rank-sum test, *P* < 0.001; Fig. 2D), alongside elevated betweenness and closeness centrality (Table S3). Phylogenetic analysis revealed that the overlapping MAGs represented 20 distinct phyla, with Pseudomonadota, Actinomycetota, and Acidobacteria representing the most abundant groups (Fig. 2E). The rhizoplane network identified 23 hub MAGs spanning 16 orders. Interestingly, these hub MAGs did not overlap with the hub MAGs in the total network. Among these, Rhizobiales was the dominant order (5 MAGs, 21.7% of total hub MAGs), followed by Chitinophagales (3 MAGs) and Pyrinomonadales (2 MAGs), while the rhizosphere network did not identify any hub MAGs.

**Fig 3:**
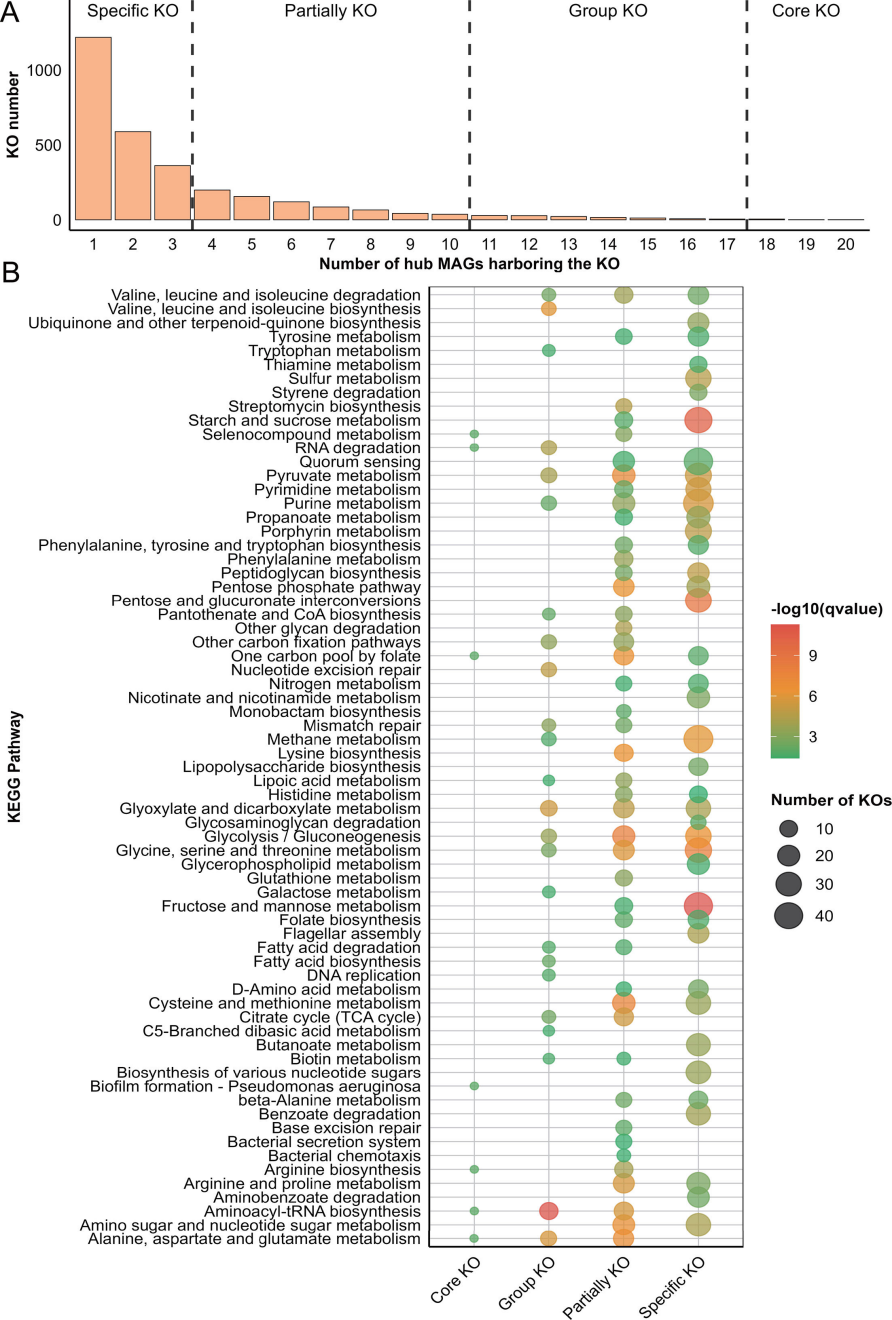
Distribution and functional enrichment of KEGG orthologs (KOs) across hub MAGs in the rhizoplane network. (**A**) Number of KOs assigned to each occurrence frequency class among hub MAGs of the rhizoplane network. KOs were classified into four categories: specific KO (present in ≤3 MAGs), partial KO (present in 4–10 MAGs), group KO (present in 11–17 MAGs), and core KO (present in ≥18 MAGs). (**B**) KEGG pathway enrichment analysis of each KO category.

To elucidate the functional potential of these hub MAGs, we annotated the genes from MAGs against the Kyoto Encyclopedia of Genes and Genomes (KEGG) database. A total of 3,003 KEGG orthologs (KOs) were identified across the hub MAGs. Based on their distribution frequency, we categorized these KOs into four tiers: 7 “core KOs” present in nearly all hub MAGs; 119 “group KOs” (present in 11–17 MAGs); 707 “partially KOs” (4–10 MAGs); and 2,170 “specific KOs” (present in fewer than 3 MAGs; Fig. 3A). KEGG enrichment analysis revealed distinct pathway associations for each group. Core and group KOs were predominantly enriched in fundamental cellular processes, such as aminoacyl-tRNA biosynthesis, alanine/aspartate/glutamate metabolism, and RNA degradation. In contrast, partial and specific KOs were more frequently associated with diverse metabolic and adaptive functions, including starch/sucrose metabolism, fructose/mannose metabolism, amino sugar/nucleotide sugar metabolism, quorum sensing, flagellar assembly, and various amino acid metabolism pathways (Fig. 3B).

In summary, the rhizosphere harbors higher microbial diversity, indicating it contains more microbial species than the rhizoplane. Moreover, the rhizoplane maintains a microbial co-occurrence network with greater complexity and connectivity than that of the rhizosphere, implying tighter microbe-microbe interactions. The hub nodes (key taxa, e.g., Rhizobiales) within this network contain genes encoding a wide range of critical functions, including metabolic processes and microbial interactions.

### Functional niche differentiation between rhizoplane and rhizosphere microbiome

**Fig 4:**
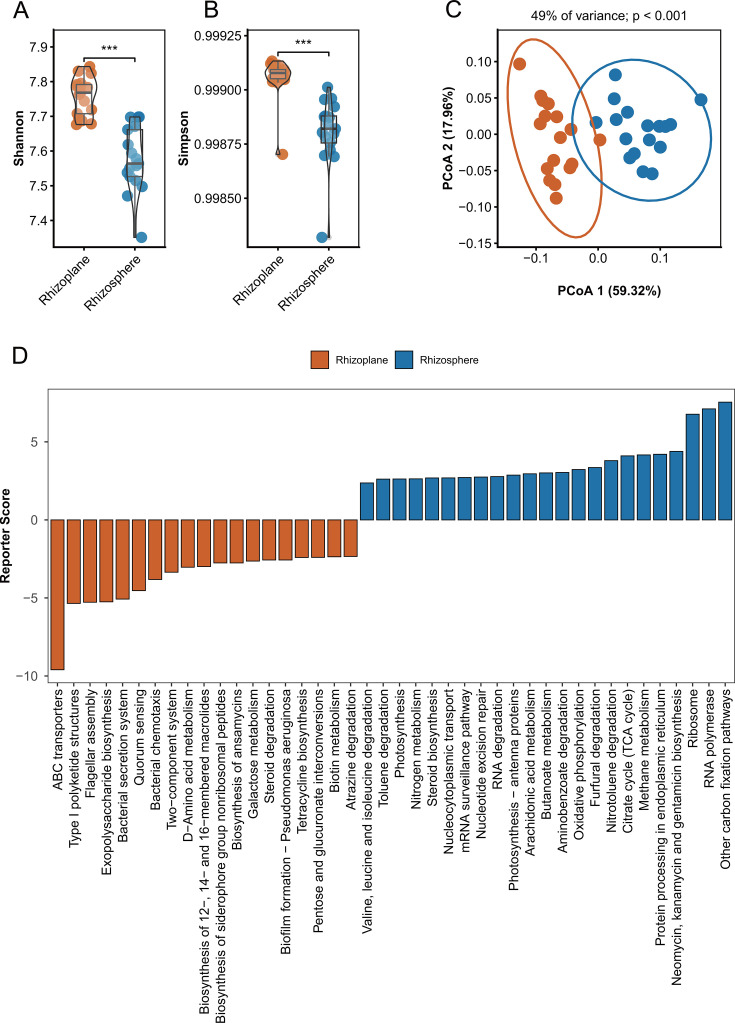
Microbial community KEGG functions from the non-redundant gene catalog in the rhizoplane and rhizosphere. (**A** and **B**) Comparison of alpha diversity (Shannon index, **A**; Simpson index, **B**) between rhizoplane (orange) and rhizosphere (blue) niches. Significance was assessed using the Wilcoxon rank-sum test (*** indicates *P* < 0.001). Box plots show the median (center line) and the 25th and 75th percentiles (box). (**C**) Principal coordinate analysis (PCoA) plot based on Bray-Curtis dissimilarity of KOs. (**D**) Reporter score analysis of microbial KEGG pathways, comparing pathway enrichment between the rhizoplane (orange) and rhizosphere (blue).

To investigate the functional distinctions between rhizoplane and rhizosphere microbiome, we conducted functional analysis based on non-redundant gene catalog derived from metagenomes. Notably, alpha diversity calculated from species profiles revealed that the rhizosphere harbored a significantly higher Shannon index than the rhizoplane across all samples (pairwise Wilcoxon rank-sum test, *P* < 0.05; Fig. S1A and B). Furthermore, microbial community composition differed substantially between the two niches (PERMANOVA, *P* < 0.001; Fig. S1C). Read-based taxonomic profiling using Kraken2 similarly demonstrated that the rhizosphere had higher Shannon and Simpson indices compared to the rhizoplane (pairwise Wilcoxon rank-sum test, *P* < 0.05; Fig. S1D and E) and a significantly distinct community composition (PERMANOVA, *P* < 0.001; Fig. S1F). Compared to the rhizosphere, the rhizoplane exhibited higher Shannon diversity (pairwise Wilcoxon rank-sum test, *P* < 0.001; Fig. 4A) and higher Simpson diversity of KOs (pairwise Wilcoxon rank-sum test, *P* < 0.001; Fig. 4B). This indicates that the diversity and evenness of functional genes (represented by KOs) carried by the rhizosphere microbial community are all comprehensively higher than those of the rhizoplane community. Significant differences in overall functional composition were confirmed between the two niches (PERMANOVA, *P* < 0.001; Fig. 4C). Generalized reporter score analysis revealed that 41 microbial functional pathways differed significantly between the rhizosphere and rhizoplane microbiome (reporter scores > 1.96, adjusted *P* value < 0.05; Fig. 4D), out of which, 19 pathways were enriched in the rhizoplane, such as ABC transporter, flagellar assembly, quorum sensing, and bacterial chemotaxis, which were observed to be enriched in rhizosphere microbiome when comparing with bulk soil microbiome in previous studies (13). The functional signature of the rhizoplane microbiome encompasses several enriched categories. Pathways for environmental sensing and signal transduction, such as quorum sensing and two-component systems, were prominent. Functions related to nutrient acquisition and transport, including ABC transporters and siderophore biosynthesis, were also enriched. In addition, pathways involved in surface colonization—namely flagellar assembly and biofilm formation—were identified. Enrichment was further observed for the biosynthesis of antimicrobial specialized metabolites, including macrolides, ansamycins, and tetracycline, as well as for catabolic pathways responsible for the degradation of steroids, galactose, and xenobiotics such as atrazine.

Meanwhile, 22 pathways were enriched in rhizosphere soil, showing the rhizosphere soil microbiome functions as a metabolic core driving biochemical cycling beyond immediate root surfaces. The rhizosphere microbiome specializes in the mineralization of complex organic matter via extensive catabolic pathways targeting aromatic compounds (e.g., toluene, nitrotoluene, and aminobenzoate) and aliphatics (e.g., butanoate and furfural). Central energy metabolism—driven by the TCA (tricarboxylic acid) cycle and oxidative phosphorylation—integrates with nitrogen transformation processes to regenerate bioavailable nutrients. Auxiliary functions, including branched-chain amino acid degradation, methane metabolism, and alternative carbon fixation pathways, further consolidate the rhizosphere’s role as the primary site for redox-driven decomposition and nutrient mobilization. This foundational activity transforms complex resources into more readily available forms, which in turn sustain the energy-intensive, direct host-microbe dialogs that are characteristic of the rhizoplane.

### Rhizoplane microbiome exhibits an enhanced capacity for nitrogen metabolism

**Fig 5:**
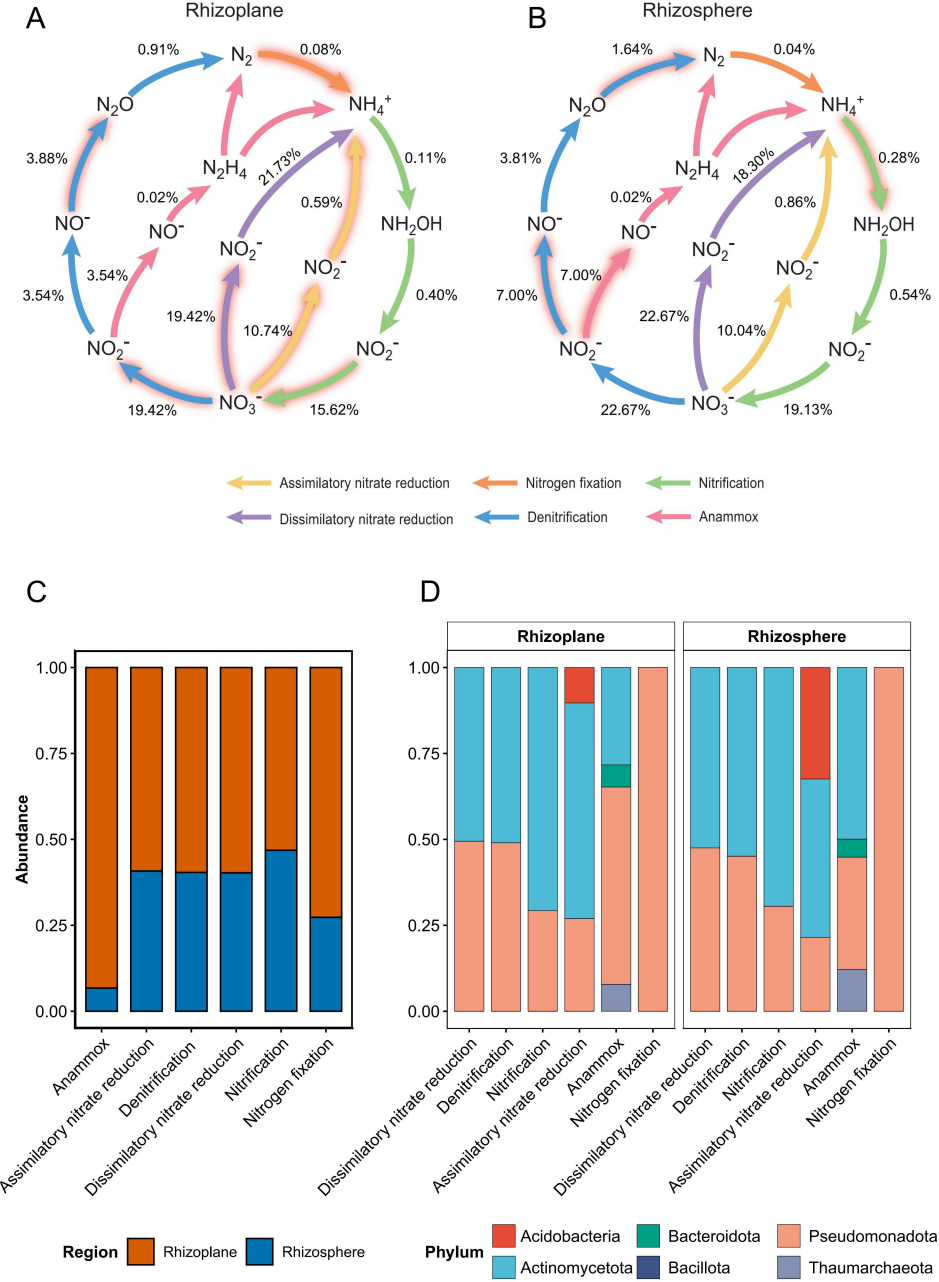
Nitrogen metabolism in the rhizoplane and rhizosphere niches. (**A** and **B**) Schematic illustration of nitrogen metabolism processes in the rhizoplane (**A**) and rhizosphere (**B**); red background shading indicates taxa that are significantly enriched in the corresponding niche. (**C**) Relative abundance of six key nitrogen metabolism genes involved in six key nitrogen metabolism processes in the rhizoplane and rhizosphere. (**D**) Taxonomic composition of each nitrogen metabolism process in the rhizoplane and rhizosphere. Colors indicate the major contributing phyla.

Nitrogen metabolism plays a critical role in plant-microbe interactions in the root zone, where biochemical transformations directly influence host nutrition and soil nutrient cycling. Among the 34 key KOs involved in nitrogen metabolism, 14 KOs were significantly enriched in the rhizoplane and 11 KOs in the rhizosphere, delineating complementary functions for each niche (pairwise Wilcoxon rank-sum test, *P* < 0.001; Table S4). Spatial partitioning of specific biochemical steps was evident across the major nitrogen transformation processes (Fig. 5A and B; Table S5). The complete reaction system of assimilatory nitrate reduction (converting NO₃⁻ to NH₄^+^ for biomass incorporation) and nitrogen fixation (converting atmospheric N₂ to bioavailable NH₄^+^) was exclusively enriched in the rhizoplane. For dissimilatory nitrate reduction, only the initial step (NO₃⁻ to NO₂⁻) was rhizoplane enriched. In contrast, a key intermediate step (NO₂⁻ to NO⁻) in the anammox process was specific to the rhizosphere. Nitrification exhibited split localization: the oxidation of NH₄^+^ to NH₂OH was rhizosphere enriched, while the subsequent oxidation of NO₂⁻ to NO₃⁻ was primarily localized to the rhizoplane. Notably, within the assimilatory nitrate reduction sequence, the first (NO₃⁻ to NO₂⁻) and third (NO⁻ to NH₄^+^) reaction steps were rhizoplane enriched, whereas the second (NO₂⁻ to NO⁻) and fourth steps (N₂O to NH₄^+^) showed rhizosphere enrichment.

This compartmentalization of enzymatic reactions reveals that the rhizosphere and rhizoplane form a synergistic, integrated nitrogen metabolic system, each specializing in specific steps to facilitate efficient nitrogen turnover at the root-soil interface. Besides, the abundance of genes originating from the rhizoplane was higher than that from the rhizosphere across six nitrogen metabolic processes (Fig. 5C; Table S6). Critically, most of the reactions culminating in plant-available ammonium (NH₄^+^) were enriched in the rhizoplane.

We further identified the microbial taxa contributing to six nitrogen metabolism processes (Fig. 5D; Table S7). The results showed that nearly all genes associated with nitrogen fixation were derived from Pseudomonadota. Those involved in assimilatory nitrate reduction originated from Pseudomonadota, Actinomycetota, and Acidobacteria, with the relative contribution of Acidobacteria being significantly higher in the rhizosphere than in the rhizoplane. For dissimilatory nitrate reduction and denitrification, Actinomycetota and Pseudomonadota each contributed approximately half of the associated genes. In nitrification, Actinomycetota accounted for about 70% of the genes. The anammox process involved genes from Pseudomonadota, Actinomycetota, Bacteroidota, and Thaumarchaeota. Notably, the proportion of Pseudomonadota in this process was markedly higher in the rhizosphere than in the rhizoplane, while the opposite was true for Actinomycetota.

### Rhizoplane microbiome exhibits an enhanced capacity for carbohydrate metabolism

**Fig 6:**
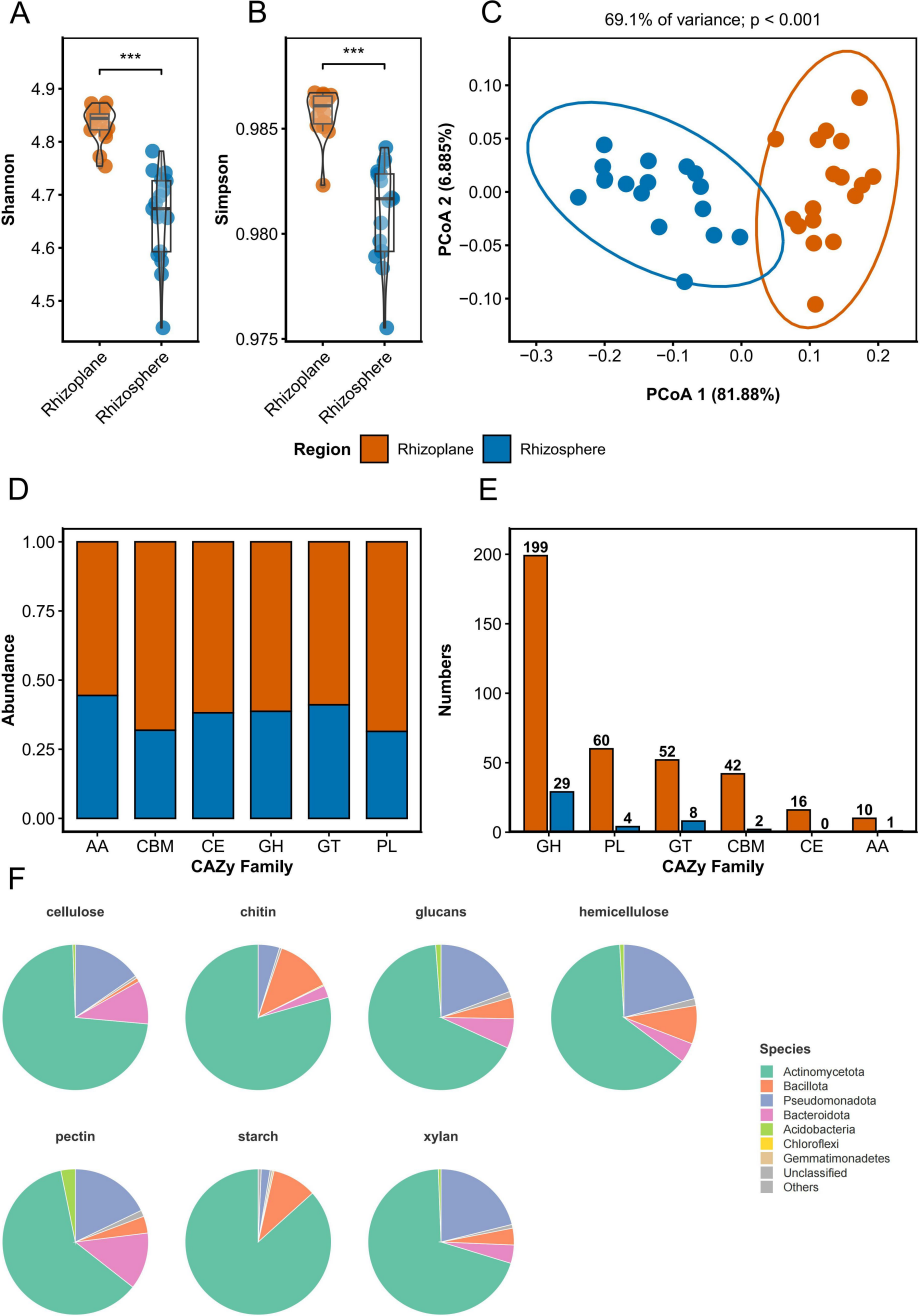
Carbohydrate-active enzyme (CAZyme) profiles in the rhizoplane and rhizosphere niches. (**A and B**) Comparison of alpha diversity (Shannon index,** A**; Simpson index, **B**) between rhizoplane (orange) and rhizosphere (blue) niches. Significance was assessed using the Wilcoxon rank-sum test (*** indicates *P* < 0.001). Box plots show the median (center line) and the 25th and 75th percentiles (box). (**C**) Principal coordinate analysis (PCoA) plot based on Bray-Curtis dissimilarity of CAZyme families. (**D**) Relative abundance of CAZyme families in the rhizoplane and rhizosphere. (**E**) Number of enriched CAZyme families in the rhizoplane and rhizosphere. (**F**) Taxonomic origins of substrates targeted by CAZyme families enriched in the rhizoplane.

To elucidate niche-specific strategies of carbohydrate utilization, we compared the abundance profiles of carbohydrate-active enzymes (CAZymes) between the rhizosphere and rhizoplane microbiome. Similar to the KEGG annotation results, the rhizoplane microbiome displayed a higher Shannon index for CAZyme family diversity compared to the rhizosphere (pairwise Wilcoxon rank-sum test, *P* < 0.001; Fig. 6A and B), indicating a more even distribution of enzyme families despite a lower estimated total richness. The overall composition of CAZyme families was also significantly distinct between the two compartments (PERMANOVA, *P* < 0.001; Fig. 6C). Aggregated across all CAZyme families, the rhizoplane exhibited a slightly higher overall gene abundance than the rhizosphere (Fig. 6D).

We identified 423 CAZyme families that were differentially abundant between the rhizosphere and rhizoplane (pairwise Wilcoxon rank-sum test, adjusted *P* < 0.05; Fig. 6E). Of these, 379 CAZyme families were significantly enriched in the rhizoplane, compared to only 44 in the rhizosphere (Table S8). The majority of differentially abundant modules belonged to glycoside hydrolase (GH) families. In total, 228 GH families showed niche-specific abundance patterns, with 199 being enriched in the rhizoplane and only 29 in the rhizosphere; and 60 polysaccharide lyase families and 52 glycosyltransferase families were significantly enriched in the rhizoplane, compared to only 4 and 8 families, respectively, in the rhizosphere.

To assess whether functional niche differentiation underpins carbohydrate metabolism in the rhizoplane, we resolved the taxonomic origins of GHs across major polysaccharide substrates (Fig. 6F). Within the rhizoplane, the taxonomic composition of GH varied substantially according to the target substrate (cellulose, chitin, glucans, hemicellulose, pectin, starch, and xylan). Actinomycetota was the dominant phylum across nearly all substrate categories. Cellulose degradation was primarily driven by Actinomycetota (73.01%), with secondary contributions from Pseudomonadota (15.37%) and Bacteroidota (9.65%). Chitin degradation also showed strong Actinomycetotal dominance (79.47%), supported by Bacillota (12.37%) and Pseudomonadota (4.74%). For glucans and hemicellulose, Actinomycetota remained predominant (66.95% and 63.82%, respectively), followed by Pseudomonadota (19.26% and 20.84%); Bacillota contributed more to hemicellulose (8.48%) than to glucans (4.71%). In pectin degradation, Actinomycetota (61.21%) was co-dominant with Pseudomonadota (17.89%) and Bacteroidota (12.57%). Starch degradation was overwhelmingly mediated by Actinomycetota (86.68%), with Bacillota (9.83%) as the only other phylum exceeding 5% contribution. For xylan, the community was dominated by Actinomycetota (69.67%) and Pseudomonadota (21.13%), with minor roles for Bacteroidota (4.07%) and Bacillota (3.71%). These results demonstrate a clear substrate-driven microbial specialization within the rhizoplane, with Actinomycetota consistently serving as the key drivers of polysaccharide decomposition across diverse carbon sources (Table S9).

### The rhizoplane microbiome is linked to foxtail millet yield

Foxtail millet phenotype is determined by both biotic and abiotic factors. Genetic variations, soil fertility, and root microbiome are underlying factors contributing to phenotype (17). To assess the specific influence of niche-specific microbial communities on yield, we modeled the relationship between millet yield traits and both taxonomic (MAG based) and functional (CAZyme and KO based) profiles of the rhizoplane and rhizosphere microbiome. The results revealed that rhizoplane communities showed consistently stronger associations with yield than the rhizosphere communities across all data types. Specifically, MAG abundance in the rhizoplane explained a significant portion of yield variance (PERMANOVA, *R*^2^ = 0.234, *P* = 3 × 10^−4^), whereas the association was weaker in the rhizosphere (PERMANOVA, *R*² = 0.127, *P* = 0.019; Fig. S2A and B). Similarly, a significant association was detected for rhizoplane CAZyme profiles (PERMANOVA, *R*^2^ = 0.163, *P* = 0.024), but not for rhizosphere profiles (PERMANOVA, *R*^2^ = 0.054, *P* = 0.438; Fig. S2C and D). The pattern held for KEGG functional profiles, with a significant relationship in the rhizoplane (PERMANOVA, *R*^2^ = 0.184, *P* = 0.017) but not in the rhizosphere (PERMANOVA, *R*^2^ = 0.059, *P* = 0.441; Fig. S2E and F). Collectively, these results underscore the rhizoplane microbiome—both its taxonomic membership and functional attributes—as a critical biological driver of yield variation in foxtail millet.

**Fig 7:**
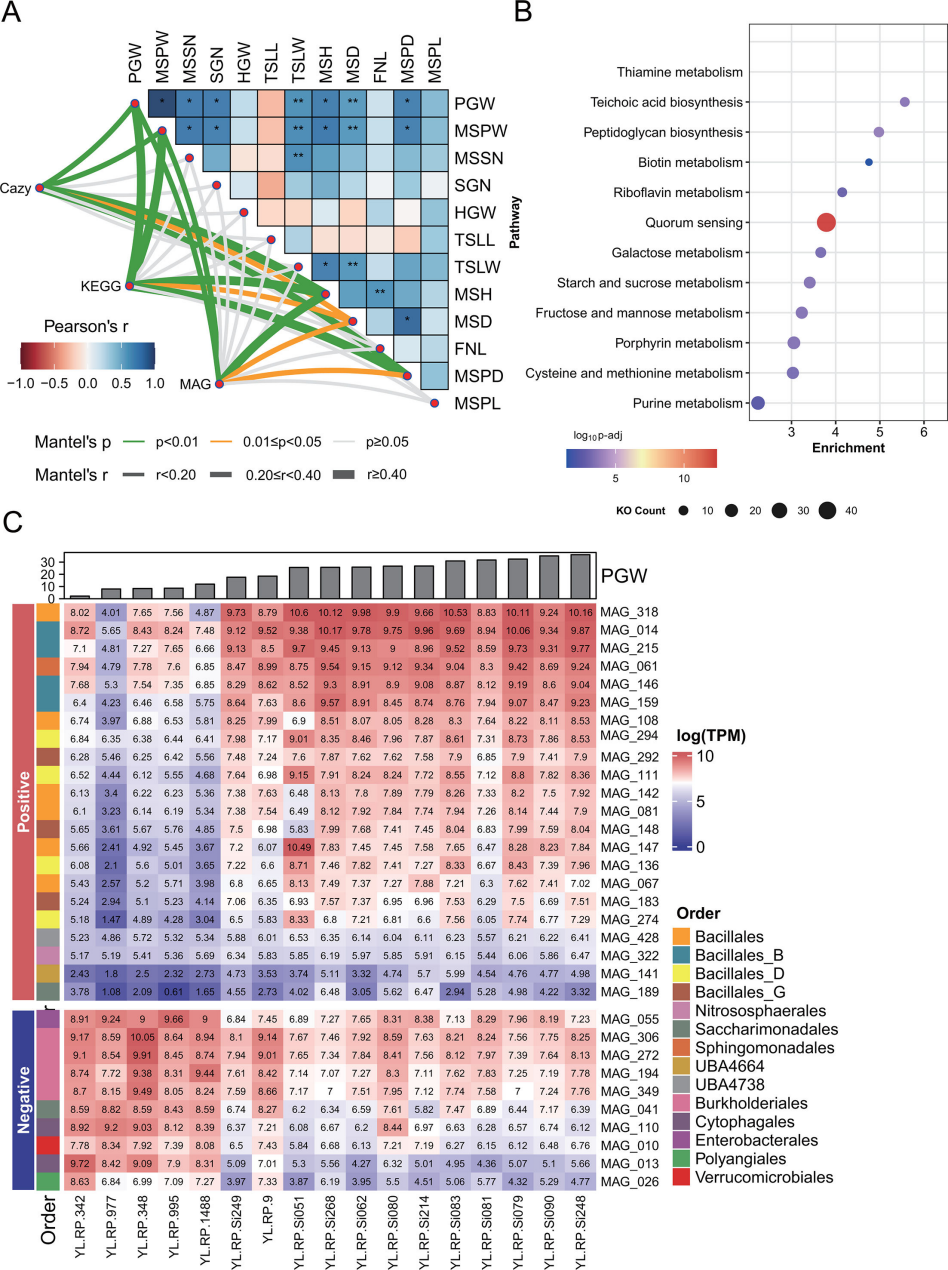
Associations between microbial functions, taxa, and foxtail millet phenotypic traits. (**A**) Mantel test correlations among CAZyme families, KEGG pathways, MAGs, and foxtail millet phenotypic traits. Correlation strength (Mantel’s *r*) and significance (*P*-value) are indicated by color intensity and asterisks, respectively. (**B**) KEGG pathway enrichment analysis of KEGG orthologs (KOs) positively correlated with foxtail millet phenotypic traits. (**C**) Abundance profiles of MAGs associated with foxtail millet phenotypic traits. MAGs are grouped by taxonomic order, and color intensity reflects relative abundance.

We next quantified 12 agronomic traits (Table S10)—top second leaf length, top second leaf width, main stem height (MSH), main stem diameter (MSD), panicle diameter of the main stem (MSPD), fringe neck length, panicle length of the main stem, per plant grain weight (PGW), main stem panicle weight (MSPW), hundred kernel weight, spikelet number of the main stem, and grain number per spike—and evaluated their correlations with rhizoplane microbial taxonomic and functional profiles. Mantel tests revealed that five traits (PGW, MSPW, MSH, MSD, and MSPD) were significantly associated with the microbial community (*P* < 0.05; Fig. 7A). Among these, PGW is a principal yield-related agronomic trait.

To identify specific microbial features linked to foxtail millet traits, we controlled for host genetic variation by incorporating genotype as a random effect in a linear mixed model. This analysis revealed that 1,342 rhizoplane KOs were positively correlated with PGW (false discovery rate [FDR] adjusted *P* < 0.05; Table S11). These KOs were functionally enriched in 13 pathways (Fig. 7B). The enrichment highlights three synergistic strategies: (i) enhanced nutritional provision to the plant, through the synthesis of essential cofactors (thiamine, biotin, and riboflavin) and the regulation of nitrogen/carbon skeletons (purine metabolism); (ii) efficient scavenging and utilization of root-derived carbon, facilitated by specialized transport (PTS, phosphotransferase system) and catabolic pathways for diverse sugars (galactose, starch/sucrose, and fructose/mannose); and (iii) robust microbial niche establishment and maintenance, supported by cell wall synthesis (peptidoglycan and teichoic acid), community coordination (quorum sensing), and stress resilience (cysteine/methionine and porphyrin metabolism). In summary, these pathways delineate a multi-functional microbial consortium that contributes to yield by enhancing plant nutrition, optimizing root carbon use, and maintaining a stable, cooperative rhizoplane microbiome.

We then identified specific CAZyme families in the rhizoplane microbiome that were associated with PGW (Table S12; Fig. S3). A total of 47 CAZyme families showed a positive correlation with yield (FDR-adjusted *P* < 0.05; Table S12; Fig. S3). Among these, several families target key plant polysaccharides: families GH13, GH30, and GH32 are involved in the hydrolysis of diverse glucans; subfamilies GH5_41, GH5_44, and GH5_47 are implicated in cellulose degradation; and families GH101, GH109, and GH59 exhibit various galactosidase activities.

We next identified specific MAGs in the rhizoplane that were associated with PGW (FDR-adjusted *P* < 0.05; Table S13; Fig. 7C). Of these, 22 MAGs—designated as plant growth-promoting rhizobacterial (PGPR) MAGs—exhibited positive correlations with millet yield, whereas 10 MAGs showed negative correlations. Among the 22 PGPR MAGs, 17 were assigned to the order Bacillales, which is well recognized to encompass numerous microbial taxa that are beneficial to plant growth and plant protection against drought-induced damage in previous research (18, 19).

We further assessed the potential promotion functions of these PGPR MAGs. KEGG annotation revealed genes encoding key enzymes for dissimilatory nitrate reduction to ammonium (*nirB*/K00362 and *nrfA*/K03385) were found in seven MAGs. Genes for alkaline phosphatases (*phoA*/K01077 and *phoD*/K01113), which mineralize organic phosphorus, were identified in 11 MAGs. Furthermore, genes encoding enzymes critical for indole-3-acetic acid (IAA) biosynthesis, including aldehyde dehydrogenase (*ALDH*/K00128) and amidase (*amiE*/K01426), were detected in 13 MAGs (Table S14). Collectively, these genetic traits suggest that the identified PGPR MAGs may enhance plant growth and yield by increasing the bioavailability of nitrogen and phosphorus and by producing the phytohormone IAA.

### Experimental validation of yield-linked PGPR MAGs

To validate the predicted plant growth-promoting effects of these PGPR MAGs on foxtail millet, we first isolated bacterial strains from the root microbiota of field-grown foxtail millet varieties and performed full-length 16S rRNA gene sequencing on these strains. We then extracted 16S rRNA gene sequences from the candidate PGPR MAGs by using barrnap (version 0.9, https://github.com/tseemann/barrnap) and aligned them with the sequences of the cultured strains. Two MAGs (MAG_081 and MAG_159, both Bacillales) shared 99.1% and 99.8% 16S rRNA gene sequence identity with their respective cultured isolates, suggesting that each pair likely represents the same species. In addition, we performed NCBI BLAST searches and found that MAG_081 showed 99.65% similarity to AF414443.1 (*Bacillus sp. YY*), and MAG_159 showed 100% identity to several *Cytobacillus* strains (e.g., PX986841.1), which was consistent with the taxonomic annotations of these MAGs at the genus level (Table S15). These two MAG-representative strains were subsequently selected for follow-up validation experiments.

**Fig 8:**
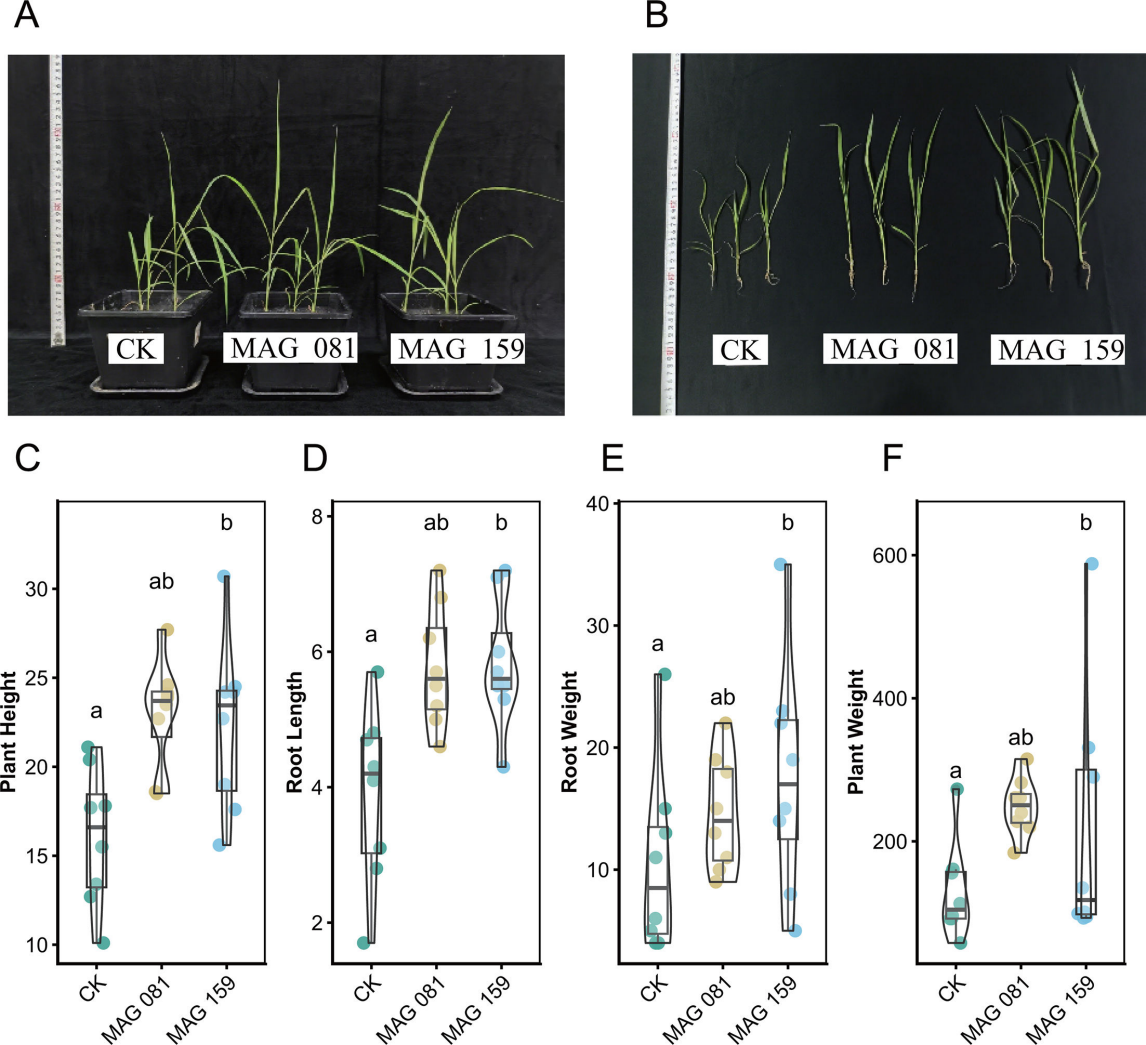
Experimental validation of PGPR MAG effects on foxtail millet phenotypic traits. (**A** and **B**) Phenotypic performance of foxtail millet inoculated with PGPR MAGs (MAG_081 and MAG_159) and an uninoculated control (CK) in pot experiments. (**C–F**) Comparison of plant height (**C**), root length (**D**), root weight (**E**), and total plant weight (**F**) of foxtail millet between the groups inoculated with PGPR MAGs and the uninoculated control.

We assessed the plant growth-promoting effects of the two candidate strains above using a pot experiment. Foxtail millet seedlings grown in sterile substrate were individually inoculated with bacterial suspensions of each strain. Consistently, the seedlings watered with suspensions of the candidate strains showed significantly increased plant height and root length compared with the control (Fig. 8A and B). Both strains improved all measured growth parameters—including plant/root length and plant/root weight—compared to the control. Notably, inoculation with the strain representing MAG_159 resulted in statistically significant increases across all these traits (Fig. 8C through F). These results confirm that the genomically identified candidate PGPR strains exhibit plant growth-promoting functions in foxtail millet. Furthermore, among the KOs previously identified as positively associated with yield, MAG_081 contained 59 KOs, and MAG_159 harbored 163 KOs. Specifically, MAG_081 possessed KOs related to nitrogen (*nirB*, K00362) and phosphorus (*phoA*, K01077) utilization, whereas MAG_159 carried genes involved in IAA biosynthesis, including aldehyde dehydrogenase (*ALDH*, K00128) and amidase (*amiE*, K01426), which may underlie its PGPR activity.

## DISCUSSION

The functional architecture of the root-associated microbiome, particularly the distinctions between the rhizosphere and rhizoplane, remains incompletely resolved, as most prior studies have relied on amplicon sequencing. This study provides a metagenomic comparison of these compartments in foxtail millet, delineating their distinct functional profiles and identifying microbial features linked to millet yield traits.

We found the rhizoplane sustains a more complex and connected microbial network than the rhizosphere, suggesting a tightly coordinated community shaped by root proximity. Functionally, the rhizoplane was enriched for pathways central to host adaptation, including flagellar assembly and chemotaxis for colonization (20); ammonium production, phosphate solubilization, and siderophore biosynthesis for nutrition (13); and specialized metabolite (e.g., tetracycline) biosynthesis for defense. In contrast, the rhizosphere was significantly enriched for pathways associated with broad biochemical cycling, including core carbon and nitrogen metabolism, as well as xenobiotic degradation, illustrating a clear functional niche differentiation. The depletion of carbon fixation pathways in the rhizoplane further implies microbial reliance on root exudates rather than inorganic carbon (21). The observed pattern of species and functional diversity suggests that the rhizoplane preferentially enriches for highly efficient microbial taxa over maximizing taxonomic diversification.

This functional specialization has direct agronomic implications. The rhizoplane’s taxonomic and functional profiles showed a stronger correlation with millet yield than those of the rhizosphere. We identified 22 yield-positive MAGs, predominantly from Bacilli, encoding genes for plant growth promotion (e.g., bioavailable nitrogen provision, phosphatase activity, and IAA synthesis). This genomic resolution advances beyond family-level associations from amplicon studies (5, 22), providing precise targets. Many Bacilli are reported to be beneficial (23), with mechanisms including drought protection via enhanced water uptake (19). Our subsequent experimental validation of cultured strains representing these MAGs confirms their plant-beneficial functions.

In summary, the rhizoplane is a functional hotspot for plant-microbe mutualism, characterized by enhanced nutrient mobilization, pathogen inhibition, and community coordination. The yield-linked microbes and functions identified here provide a genomic resource for developing microbial applications or for informing breeding programs aimed at harnessing the root microbiome to enhance sustainable crop productivity.

## MATERIALS AND METHODS

### Sample collections and DNA extraction

Foxtail millet seeds were sown in the field in Zhangjiakou (Hebei, China) and Yangling (Shaanxi, China) separately, with 20 repeats for each line. For each cultivar, the root samples from three individuals were harvested during the ripening stage and separated into rhizosphere and rhizoplane samples using an established protocol as in our previous study (5). Two bulk soil samples (one from Zhangjiakou and one from Yangling) were collected from unplanted sites far away from the foxtail millets. All the samples were placed in 15 mL clean microcentrifuge tubes and transferred back to the lab under −20°C.

Rhizosphere samples were collected by scraping loosely attached soil from the root surface, and 363 rhizoplane samples were collected from tightly adhering soil washed off by vortexing for 30 min with a mixture of 1 × PBS and the adsorbent Silwet-77. The DNA extraction procedure was done according to the protocols of the MoBio PowerSoil toolkit. Unfortunately, several samples failed to extract DNA from the rhizosphere or rhizoplanes due to low DNA concentrations. Consequently, we obtained 2 DNA samples from bulk soil, 17 DNA samples from rhizosphere, and 17 DNA samples from rhizoplanes, of which rhizosphere and rhizoplane samples were paired. The productivity trait (here referring to the grain weight per plant) was measured by weighing all the grains on each plant. Then the productivity trait was grouped into four ranks (I: <10 g, II: 11–20 g, III: 21 ~ 30 g, and IV: 31–40 g).

### Library construction for Hiseq2500 and BGISEQ500 platforms

Four rhizosphere samples (two from Zhangjiakou and two from Yangling) and two bulk soil samples (one from Zhangjiakou and one from Yangling) were prepared for sequencing by Hiseq2500. To obtain sufficient sequencing depths to capture most of the microbial genomes, seven libraries were generated for each sample. One microgram DNA was sheared to 350 bp fragments using the Covaris LE220 (Covaris, Inc., Woburn, MA, USA), size selected using AMPure XP beads (Beckman Coulter, Brea, CA, USA). Adapters were then ligated to both ends of the fragments. Fragment sizes for all libraries were measured using a LabChip GX Touch Hi Sens, and qPCR was performed on a QuantStudio 6 Flex Real-Time PCR System (Applied Biosystems) with the Kapa library quantification kit. Each barcoded library was pooled in equal amounts for each lane.

A total of 34 libraries, including 17 rhizoplane and 17 rhizosphere paired samples from Yangling, were constructed to be sequenced using the BGISEQ500 platform. A total of 500 ng of input DNA was used for library formation and fragmented ultrasonically with Covaris E220 (Covaris, Brighton, UK), yielding 400–600 bp fragments. Sheared DNA without size selection was purified with an AxygenTMAxyPrepTM Mag PCR Clean-Up Kit. An equal volume of beads was added to each sample, and DNA was eluted with 45 μL TE buffer. We performed end-repairing and A-tailing with a 2:2:1 mixture of T4 DNA polymerase (ENZYMATICSTM P708–1500), T4 polynucleotide kinase (ENZYMATICSTM Y904–1500), and rTaq DNA polymerase (TAKARATM R500Z). A total of 20 ng of purified DNA was used, and enzymes were heat inactivated at 75°C. Adaptors with specific barcodes (Ad153 2B) were ligated to the DNA fragment by T4 DNA ligase (ENZYMATICSTM L603-HC-1500) at 23°C. After the ligation, PCR amplification was carried out. A total of 55 ng of purified PCR products were denatured at 95°C and ligated by T4 DNA ligase (ENZYMATICSTM L603-HC-1500) at 37°C to generate a single-strand circular DNA library. Four barcoded libraries were pooled in equal amounts to make DNA Nanoballs.

### Benchmarking CD-HIT and MMseqs2 for constructing non-redundant gene catalogs

To optimize the construction of non-redundant gene catalogs, we compared CD-HIT and MMseqs2 with sequence identity >95 and coverage >90 (24–27) with CD-HIT (28). Despite yielding near-identical catalog sizes (104.15 vs. 103.80 million genes) and 91% gene content overlap, MMseqs2 proved dramatically more efficient, processing data ~30 times faster (59,368 vs. 1,799,100 CPU seconds). This demonstrates MMseqs2’s superior efficiency for large-scale metagenomic clustering without compromising output quality. Leveraging the deep metagenomic sequencing data from two bulk soil and four rhizosphere samples collected from geographically distinct sites, we constructed six sample-specific non-redundant gene catalogs. Each catalog contained approximately 30 million non-redundant genes. Then we estimated the effect of sequencing depths on gene recovery. Similar saturation patterns were observed across both bulk soil and rhizosphere samples. Gene discovery curves approached saturation when utilizing the full data set for each sample, indicating that nearly the complete gene repertoire was captured. Specifically, in rhizosphere samples, sequencing 10 Gb of data recovered approximately 11 million genes, whereas 40 Gb of data yielded about 23 million genes, representing roughly 70% of the total genes in the respective sample-specific catalog.

### Metagenomic sequencing, assembly, and binning

Four rhizosphere samples (two from Zhangjiakou and two from Yangling) and two bulk soil samples (one from Zhangjiakou and one from Yangling) were deeply sequenced on the Illumina HiSeq2500 platform using a paired-end 150 bp sequencing strategy and V4 reagents, and the remaining 50 samples were sequenced by BGISEQ500 using a paired-end 100 bp sequencing strategy. Raw reads were filtered by SOAPnuke (v1.5.6) (29) with parameters set as “filterMeta -Q 2 -S -L 15 -N 3 -P 0.5 -q 20 -l 60 -R 0.5-5 0.” We obtained an average of 322 ± 13.8 Gb of clean data for each HiSeq-sequenced sample and 33 ± 6.5 Gb of clean data for each BGISEQ-sequenced sample. We assumed that more sequencing data would improve the assembly of metagenomic reads, so six samples sequenced on the HiSeq platform were co-assembled by Megahit-1.0.3 (30); the rest of the samples were assembled separately. Assembled contigs with lengths less than 500 bp were discarded for the following analyses.

All the resulting assemblies were subsequently individually clustered into genomic MAGs. First, reads from all assemblies were separately cross-mapped to all scaffolds >2 kb in size from a single assembly using Bowtie2 to generate a coverage profile for the scaffolds of that assembly across all samples. Scaffold differential coverage profiles were input into software MetaBAT2 (31), CONCOCT (32), and MaxBin (33) to run binning on all samples individually. Then CheckM (34) was used to assess the completeness and contamination of all MAGs, filtering for completeness ≥70% and contamination ≤10% or completeness ≥50% and contamination ≤5%. To obtain non-redundant MAGs, dereplication was performed using dRep (35). The dereplication workflow was used with options: dRep dereplicate outdir2 -g --S_algorithm gANI -nc 0.6 –noQualityFiltering. A final set of 595 non-redundant MAGs was obtained. Taxonomic assignment of these MAGs was performed using GTDB-Tk release 207 (36) with the option: gtdbtk classify_wf --cpus 20 –genome --extension fa.

### Non-redundant gene set construction

The non-redundant gene set was usually used for characterizing the genetic potential of ecologically complex environments, especially the number of unique functional genes. We constructed a unique gene set using amino acid sequences by MMseqs2. Genes were predicted over the contigs assembled from each test sample by Prodigal (37) under the “Meta” mode, and only genes with a length greater than or equal to 500 bp were retained. All of the nucleic acid sequences of genes were merged and input into MMseqs2 (38) with 0.9 coverage and 0.95 identity to generate the non-redundant gene catalog.

### Functional and taxonomic annotations

Functional annotations were implemented by mapping non-redundant genes against KEGG (version 93) (39) by Diamond (40), and alignment results meeting the criteria: hit score > 60, win score = 1, were retained. Taxonomic annotations were executed by mapping genes to the NCBI “nr” database with Diamond, and alignments were filtered with coverage ≥ 0.8 and identity ≥ 0.65. Taxonomic information was obtained using an in-house lowest common ancestor pipeline (41). In brief, if the taxonomy of each retained hit for a query gene is consistent under certain taxonomic levels, then the consistent taxonomic information is added to that gene.

Cazymes were annotated by searching genes against the CAZy database (42) hmm profiles provided by dbCAN (43), and the results were filtered by the embedded pipeline in dbCAN.

### Gene abundance profiling

The transcripts per million (TPM) metric was adopted to quantify the relative abundance of metagenomic genes. For each gene, the TPM value was calculated by mapping clean reads against the gene catalog using Salmon (v1.9.0) (44) in “meta” mode, as the following formula:


$$\text{TPM}=(N_{i}/L_{i})\times 1,000,000/\sum (N_{i}/L_{i}+\cdots +N_{m}/L_{m}).$$

Subsequently, the TPM values of all genes across all samples were compiled into a comprehensive gene abundance matrix.

KO abundances were obtained by summing the TPM values of genes annotated to the same KO. Similarly, CAZyme family abundances were obtained by summing the TPM values of genes annotated to the same family; the results were compiled into a CAZyme family abundance table.

### Co-abundance network analysis

Microbial co-abundance networks were constructed using MAGs. Networks were built using the “Microeco” R package (v1.14.0) (45) as follows: (i) Spearman rank correlation coefficients (ρ) between all feature pairs were computed using trans_network$new; (ii) optimal correlation thresholds for network construction were determined using cal_network (COR_optimization = TRUE). Network topology properties and node centrality measures for all networks were calculated using the “ggClusterNet” R package (46). Hub nodes within these networks were identified via Zi-Pi analysis (Zi ≥ 2.5 and Pi < 0.62, Module hubs; Zi < 2.5 and Pi ≥ 0.62, Connectors; Zi ≥ 2.5 and Pi ≥ 0.62, Network hubs). Then, the network was visualized using the hierarchical tree map algorithm (model_maptree2) from ggClusterNet package. Finally, the network was visualized by Gephi (47).

### Statistical analyses

Shannon and Simpson indices are calculated using diversity() function of “vegan” package (48) defined as:


$$\begin{array}{c} \text{ Shannon: }H=-\sum_{i=1}^{S}p_{i}\log_{b}p_{i}\\ \text{ Simpson: }D=1-\sum_{i=1}^{S}p_{i}^{2} \end{array}$$

where *p*_*i*_ is the proportional abundance of species *i*, *S* is the number of species, and *b* is the base of the logarithm.

Comparisons of alpha diversity between groups were performed by the pairwise Wilcoxon rank-sum test via the wilcox_test() function of the “rstatix” package.

The beta diversity on microbial and functional Bray-Curtis dissimilarity was tested with PERMANOVA using the Adonis2 function in R package “vegan.” Data visualizations were primarily generated using the “ggplot2” R package (49).

Comparisons between groups of KOs, CAZymes, and MAGs were performed by the pairwise Wilcoxon rank-sum test via the wilcox_test() function of the “rstatix” package.

Significant KOs, CAZymes, and MAGs correlating with yield were calculated using the linear mixed-effect model in lme4 package (50) setting yield as a fixed effect and host genotype clusters as a random effect, and the *P*-values were adjusted using “FDR” method.

### Generalized reporter score analysis

We employed the ReporterScore package (51) for Generalized Reporter Score-based Enrichment Analysis. The abundances of KOs were tested for enrichment and depletion between different group. *P* values were adjusted by the FDR method. In brief, a one-tailed Wilcoxon rank-sum test was performed on all the KOs and adjusted for multiple testing using the Benjamini-Hochberg method. The *Z*-score for each KO could then be calculated:


$$Z_{{\text{KO}}_{i}}=\Theta^{-1}(1-P_{{\text{KO}}_{i}}),$$

where Ɵ^−1^ is the inverse normal cumulative distribution, *P*_KO*i*_ is the adjusted *P* value for that KO. The reporter score for a KEGG pathway is then:


$$\begin{array}{c} Z_{\text{pathway }}=\frac{1}{\sqrt{k}}\sum Z_{\text{KOi }}\\ \text{ Reporter score }=\frac{Z_{\text{pathway }}-\mu_{k}}{\sigma_{k}} \end{array}$$

where *k* is the number of KOs involved in the pathway.

### Isolation of root-derived bacteria

All of the soil samples were stored at −20°C and immediately taken back to the laboratory for further preparation. The root samples were cut into small sections of 2–3 cm, put into a 2 mL Eppendorf tube containing 1.5 mL sterile PBS-S buffer, and washed on a shaking platform for 20 min at 25 r/s. The washing buffer was then subjected to centrifugation for 5 min at 137 × *g*. The supernatants were diluted from 10^−1^ to 10^−7^, and then 10^−4^ and 10^−6^ dilutions were distributed and cultivated in 96-well microtiter plates in Nutrient Agar media (BD Company) for 48–72 h at 28°C. The full length of the 16S rRNA gene for each strain was amplified with 16S universal primers (27 F 5′-GAGTTTGATCCTGGCTCAG-3′; 1492 R 5′-TACCTTGTTACGACTT-3′).

### Plant growth promotion assay

To validate the promoting effect of PGPR MAGs on foxtail growth, two MAGs with representative strains were selected for the validation experiment, including MAG_081 and MAG_159. To further validate the promoting or suppressing effect of marker microbes on foxtail millet growth conditions, we sowed the germfree Huagu12 seeds into a small basin with the sterilized soil collected from the planting field of foxtail millet in the greenhouse of CNGB, Shenzhen. Then the suspension of marker strain was used to water the 5-day-old seedlings in the basin, with three repeats for each strain. The control was watered with sterilized water. After 14 days of inoculation, the plant height and root length were measured and assessed by Dunn’s Multiple Comparison Test with *P*-values adjusted by the FDR method.

## Data Availability

The scripts for in this work are available on GitHub (https://github.com/jincanzhi/millet-rhizoplane-microbiome). The and rhizosphere metagenomes data that support the findings of this study have been deposited into CNSA with accession number CNP0004455.

## SUPPLEMENTAL MATERIAL

Supplemental figures spectrum.00707-26-s0001.docx Figures S1 to S3.

Supplemental tables spectrum.00707-26-s0002.xlsx Tables S1 to S15.

ASM does not own the copyrights to Supplemental Material that may be linked to, or accessed through, an article. The authors have granted ASM a non-exclusive, world-wide license to publish the Supplemental Material files. Please contact the corresponding author directly for reuse.

## References

[1] Levy A, Salas Gonzalez I, Mittelviefhaus M, Clingenpeel S, Herrera Paredes S, Miao J, Wang K, Devescovi G, Stillman K, Monteiro F, et al.. 2017. Genomic features of bacterial adaptation to plants. Nat Genet 50:138–150. doi:10.1038/s41588-017-0012-9 29255260 PMC5957079

[2] Philippot L, Raaijmakers JM, Lemanceau P, van der Putten WH. 2013. Going back to the roots: the microbial ecology of the rhizosphere. Nat Rev Microbiol 11:789–799. doi:10.1038/nrmicro3109 24056930

[3] Bulgarelli D, Schlaeppi K, Spaepen S, Ver Loren van Themaat E, Schulze-Lefert P. 2013. Structure and functions of the bacterial microbiota of plants. Annu Rev Plant Biol 64:807–838. doi:10.1146/annurev-arplant-050312-120106 23373698

[4] Edwards J, Johnson C, Santos-Medellín C, Lurie E, Podishetty NK, Bhatnagar S, Eisen JA, Sundaresan V. 2015. Structure, variation, and assembly of the root-associated microbiomes of rice. Proc Natl Acad Sci USA 112. doi:10.1073/pnas.1414592112 PMC4345613 25605935

[5] Jin T, Wang Y, Huang Y, Xu J, Zhang P, Wang N, Liu X, Chu H, Liu G, Jiang H, et al.. 2017. Taxonomic structure and functional association of foxtail millet root microbiome. Gigascience 6:1–12. doi:10.1093/gigascience/gix089 PMC7059795 29050374

[6] Peiffer JA, Spor A, Koren O, Jin Z, Tringe SG, Dangl JL, Buckler ES, Ley RE. 2013. Diversity and heritability of the maize rhizosphere microbiome under field conditions. Proc Natl Acad Sci USA 110:6548–6553. doi:10.1073/pnas.1302837110 23576752 PMC3631645

[7] Bulgarelli D, Rott M, Schlaeppi K, Ver Loren van Themaat E, Ahmadinejad N, Assenza F, Rauf P, Huettel B, Reinhardt R, Schmelzer E, Peplies J, Gloeckner FO, Amann R, Eickhorst T, Schulze-Lefert P. 2012. Revealing structure and assembly cues for *Arabidopsis* root-inhabiting bacterial microbiota. Nature 488:91–95. doi:10.1038/nature11336 22859207

[8] Zarraonaindia I, Owens SM, Weisenhorn P, West K, Hampton-Marcell J, Lax S, Bokulich NA, Mills DA, Martin G, Taghavi S, van der Lelie D, Gilbert JA. 2015. The soil microbiome influences grapevine-associated microbiota. mBio 6:e02527-14. doi:10.1128/mBio.02527-14 25805735 PMC4453523

[9] Bulgarelli D, Garrido-Oter R, Münch PC, Weiman A, Dröge J, Pan Y, McHardy AC, Schulze-Lefert P. 2015. Structure and function of the bacterial root microbiota in wild and domesticated barley. Cell Host Microbe 17:392–403. doi:10.1016/j.chom.2015.01.011 25732064 PMC4362959

[10] Vejan P, Abdullah R, Khadiran T, Ismail S, Nasrulhaq Boyce A. 2016. Role of plant growth promoting rhizobacteria in agricultural sustainability-a review. Molecules 21:573. doi:10.3390/molecules21050573 27136521 PMC6273255

[11] Xu J, Zhang Y, Zhang P, Trivedi P, Riera N, Wang Y, Liu X, Fan G, Tang J, Coletta-Filho HD, et al.. 2018. The structure and function of the global citrus rhizosphere microbiome. Nat Commun 9. doi:10.1038/s41467-018-07343-2 PMC6244077 30459421

[12] Knief C, Delmotte N, Chaffron S, Stark M, Innerebner G, Wassmann R, von Mering C, Vorholt JA. 2012. Metaproteogenomic analysis of microbial communities in the phyllosphere and rhizosphere of rice. ISME J 6:1378–1390. doi:10.1038/ismej.2011.192 22189496 PMC3379629

[13] Ofek-Lalzar M, Sela N, Goldman-Voronov M, Green SJ, Hadar Y, Minz D. 2014. Niche and host-associated functional signatures of the root surface microbiome. Nat Commun 5:4950. doi:10.1038/ncomms5950 25232638

[14] Zhang Y, Xu J, Riera N, Jin T, Li J, Wang N. 2017. Huanglongbing impairs the rhizosphere-to-rhizoplane enrichment process of the citrus root-associated microbiome. Microbiome 5:97. doi:10.1186/s40168-017-0304-4 28797279 PMC5553657

[15] He L, Zhang B, Wang X, Li H, Han Y. 2015. Foxtail millet: nutritional and eating quality, and prospects for genetic improvement. Front Agr Sci Eng 2:124. doi:10.15302/J-FASE-2015054

[16] Jia G, Liu X, Schnable JC, Niu Z, Wang C, Li Y, Wang S, Wang S, Liu J, Guo E, Zhi H, Diao X. 2015. Microsatellite variations of elite setaria varieties released during last six decades in China. PLoS One 10:e0125688. doi:10.1371/journal.pone.0125688 25932649 PMC4416935

[17] Lian Y, Meng X, Yang Z, Wang T, Ali S, Yang B, Zhang P, Han Q, Jia Z, Ren X. 2017. Strategies for reducing the fertilizer application rate in the ridge and furrow rainfall harvesting system in semiarid regions. Sci Rep 7:2644. doi:10.1038/s41598-017-02731-y 28572666 PMC5454006

[18] Wang Y, Wang X, Sun S, Jin C, Su J, Wei J, Luo X, Wen J, Wei T, Sahu SK, Zou H, Chen H, Mu Z, Zhang G, Liu X, Xu X, Gram L, Yang H, Wang E, Liu H. 2022. GWAS, MWAS and mGWAS provide insights into precision agriculture based on genotype-dependent microbial effects in foxtail millet. Nat Commun 13. doi:10.1038/s41467-022-33238-4 PMC9546826 36207301

[19] Marulanda A, Barea J-M, Azcón R. 2009. Stimulation of plant growth and drought tolerance by native microorganisms (AM fungi and bacteria) from dry environments: mechanisms related to bacterial effectiveness. J Plant Growth Regul 28:115–124. doi:10.1007/s00344-009-9079-6

[20] Schulz-Bohm K, Gerards S, Hundscheid M, Melenhorst J, de Boer W, Garbeva P. 2018. Calling from distance: attraction of soil bacteria by plant root volatiles. ISME J 12:1252–1262. doi:10.1038/s41396-017-0035-3 29358736 PMC5931972

[21] Bais HP, Weir TL, Perry LG, Gilroy S, Vivanco JM. 2006. The role of root exudates in rhizosphere interactions with plants and other organisms. Annu Rev Plant Biol 57:233–266. doi:10.1146/annurev.arplant.57.032905.105159 16669762

[22] Song WY, Hwang CY, Schaad NW. 2004. Detection of *Acidovorax avenae* ssp. *avenae* in rice seeds using BIO‐PCR. J Phytopathol 152:667–676. doi:10.1111/j.1439-0434.2004.00914.x

[23] Radhakrishnan R, Hashem A, Abd Allah EF. 2017. *Bacillus*: a biological tool for crop improvement through bio-molecular changes in adverse environments. Front Physiol 8:667. doi:10.3389/fphys.2017.00667 28932199 PMC5592640

[24] Li J, Jia H, Cai X, Zhong H, Feng Q, Sunagawa S, Arumugam M, Kultima JR, Prifti E, Nielsen T, et al.. 2014. An integrated catalog of reference genes in the human gut microbiome. Nat Biotechnol 32:834–841. doi:10.1038/nbt.2942 24997786

[25] Sunagawa S, Coelho LP, Chaffron S, Kultima JR, Labadie K, Salazar G, Djahanschiri B, Zeller G, Mende DR, Alberti A, et al.. 2015. Structure and function of the global ocean microbiome. Science 348:1261359. doi:10.1126/science.1261359 25999513

[26] Xiao L, Feng Q, Liang S, Sonne SB, Xia Z, Qiu X, Li X, Long H, Zhang J, Zhang D, et al.. 2015. A catalog of the mouse gut metagenome. Nat Biotechnol 33:1103–1108. doi:10.1038/nbt.3353 26414350

[27] Xiao L, Estellé J, Kiilerich P, Ramayo-Caldas Y, Xia Z, Feng Q, Liang S, Pedersen AØ, Kjeldsen NJ, Liu C, Maguin E, Doré J, Pons N, Le Chatelier E, Prifti E, Li J, Jia H, Liu X, Xu X, Ehrlich SD, Madsen L, Kristiansen K, Rogel-Gaillard C, Wang J. 2016. A reference gene catalogue of the pig gut microbiome. Nat Microbiol 1:16161. doi:10.1038/nmicrobiol.2016.161 27643971

[28] Li W, Godzik A. 2006. Cd-hit: a fast program for clustering and comparing large sets of protein or nucleotide sequences. Bioinformatics 22:1658–1659. doi:10.1093/bioinformatics/btl158 16731699

[29] Chen Y, Chen Y, Shi C, Huang Z, Zhang Y, Li S, Li Y, Ye J, Yu C, Li Z, Zhang X, Wang J, Yang H, Fang L, Chen Q. 2018. SOAPnuke: a MapReduce acceleration-supported software for integrated quality control and preprocessing of high-throughput sequencing data. Gigascience 7:1–6. doi:10.1093/gigascience/gix120 PMC5788068 29220494

[30] Li D, Liu C-M, Luo R, Sadakane K, Lam T-W. 2015. MEGAHIT: an ultra-fast single-node solution for large and complex metagenomics assembly via succinct de Bruijn graph. Bioinformatics 31:1674–1676. doi:10.1093/bioinformatics/btv033 25609793

[31] Kang DD, Froula J, Egan R, Wang Z. 2015. MetaBAT, an efficient tool for accurately reconstructing single genomes from complex microbial communities. PeerJ 3:e1165. doi:10.7717/peerj.1165 26336640 PMC4556158

[32] Alneberg J, Bjarnason BS, de Bruijn I, Schirmer M, Quick J, Ijaz UZ, Lahti L, Loman NJ, Andersson AF, Quince C. 2014. Binning metagenomic contigs by coverage and composition. Nat Methods 11:1144–1146. doi:10.1038/nmeth.3103 25218180

[33] Wu Y-W, Simmons BA, Singer SW. 2016. MaxBin 2.0: an automated binning algorithm to recover genomes from multiple metagenomic datasets. Bioinformatics 32:605–607. doi:10.1093/bioinformatics/btv638 26515820

[34] Parks DH, Imelfort M, Skennerton CT, Hugenholtz P, Tyson GW. 2015. CheckM: assessing the quality of microbial genomes recovered from isolates, single cells, and metagenomes. Genome Res 25:1043–1055. doi:10.1101/gr.186072.114 25977477 PMC4484387

[35] Olm MR, Brown CT, Brooks B, Banfield JF. 2017. dRep: a tool for fast and accurate genomic comparisons that enables improved genome recovery from metagenomes through de-replication. ISME J 11:2864–2868. doi:10.1038/ismej.2017.126 28742071 PMC5702732

[36] Parks DH, Chuvochina M, Waite DW, Rinke C, Skarshewski A, Chaumeil P-A, Hugenholtz P. 2018. A standardized bacterial taxonomy based on genome phylogeny substantially revises the tree of life. Nat Biotechnol 36:996–1004. doi:10.1038/nbt.4229 30148503

[37] Hyatt D, Chen G-L, Locascio PF, Land ML, Larimer FW, Hauser LJ. 2010. Prodigal: prokaryotic gene recognition and translation initiation site identification. BMC Bioinformatics 11:119. doi:10.1186/1471-2105-11-119 20211023 PMC2848648

[38] Steinegger M, Söding J. 2017. MMseqs2 enables sensitive protein sequence searching for the analysis of massive data sets. Nat Biotechnol 35:1026–1028. doi:10.1038/nbt.3988 29035372

[39] Kanehisa M, Furumichi M, Tanabe M, Sato Y, Morishima K. 2017. KEGG: new perspectives on genomes, pathways, diseases and drugs. Nucleic Acids Res 45:D353–D361. doi:10.1093/nar/gkw1092 27899662 PMC5210567

[40] Buchfink B, Reuter K, Drost H-G. 2021. Sensitive protein alignments at tree-of-life scale using DIAMOND. Nat Methods 18:366–368. doi:10.1038/s41592-021-01101-x 33828273 PMC8026399

[41] Huson DH, Auch AF, Qi J, Schuster SC. 2007. MEGAN analysis of metagenomic data. Genome Res 17:377–386. doi:10.1101/gr.5969107 17255551 PMC1800929

[42] Drula E, Garron M-L, Dogan S, Lombard V, Henrissat B, Terrapon N. 2022. The carbohydrate-active enzyme database: functions and literature. Nucleic Acids Res 50:D571–D577. doi:10.1093/nar/gkab1045 34850161 PMC8728194

[43] Yin Y, Mao X, Yang J, Chen X, Mao F, Xu Y. 2012. dbCAN: a web resource for automated carbohydrate-active enzyme annotation. Nucleic Acids Res 40:W445–W451. doi:10.1093/nar/gks479 22645317 PMC3394287

[44] Patro R, Duggal G, Love MI, Irizarry RA, Kingsford C. 2017. Salmon provides fast and bias-aware quantification of transcript expression. Nat Methods 14:417–419. doi:10.1038/nmeth.4197 28263959 PMC5600148

[45] Liu C, Cui Y, Li X, Yao M. 2021. Microeco: an R package for data mining in microbial community ecology. FEMS Microbiol Ecol 97:fiaa255. doi:10.1093/femsec/fiaa255 33332530

[46] Wen T, Xie P, Yang S, Niu G, Liu X, Ding Z, Xue C, Liu Y-X, Shen Q, Yuan J. 2022. ggClusterNet: an R package for microbiome network analysis and modularity-based multiple network layouts. iMeta 1:e32. doi:10.1002/imt2.32 38868720 PMC10989811

[47] Mathieu Bastian SH. 2009. Gephi: an open source software for exploring and manipulating networks.

[48] Jari Oksanen GLS, Guillaume Blanchet F, Roeland K, Pierre L, Peter R. M. 2016. vegan: community ecology package. R package version 2.4-1.

[49] Wickham H. 2011. ggplot2: elegant graphics for data analysis.

[50] Douglas Bates MM, Bolker B, Walker S. 2025. Linear mixed-effects models using “Eigen” and S4.

[51] Peng C, Chen Q, Tan S, Shen X, Jiang C. 2024. Generalized reporter score-based enrichment analysis for omics data. Brief Bioinform 25. doi:10.1093/bib/bbae116 PMC10976918 38546324

