# Supplementary material for: The functional structure of foxtail millet rhizoplane microbiome and its association with yield
Source: Microbiol Spectr. 2026 Jul 17;14(8):e00707-26. doi: 10.1128/spectrum.00707-26 (PMC13436336; doi:10.1128/spectrum.00707-26)
Supplement: Supplementary file 1 [file spectrum.00707-26-s0001.docx]

**Supplement Figure 1. Microbial taxonomic composition based on non-redundant genes and sequencing reads in the rhizoplane and rhizosphere.**

(A, B) Comparison of alpha diversity (Shannon index, A; Simpson index, B) of gene-based taxonomic profiles between rhizoplane (orange) and rhizosphere (blue) niches. Significance was assessed using the Wilcoxon rank-sum test (* indicates p < 0.05). Box plots show the median (center line) and the 25th and 75th percentiles (box).

(C) Principal coordinate analysis (PCoA) plot based on Bray–Curtis dissimilarity of gene-derived species composition.

(D, E) Comparison of alpha diversity (Shannon index, D; Simpson index, E) of read-based taxonomic profiles between rhizoplane (orange) and rhizosphere (blue) niches. Significance was assessed using the Wilcoxon rank-sum test (** indicates p < 0.01 and * indicates p < 0.05). Box plots show the median (center line) and the 25th and 75th percentiles (box).

(F) Principal coordinate analysis (PCoA) plot based on Bray–Curtis dissimilarity of read-derived species composition.

**Supplement Figure 2. Microbial community differentiation across taxonomic and functional domains.**

(A, B) Principal coordinate analysis (PCoA) plots based on Bray-Curtis dissimilarity of MAGs for rhizoplane (A) and rhizosphere (B) samples.

(C, D) Principal coordinate analysis (PCoA) plots based on Bray-Curtis dissimilarity of CAZyme families for rhizoplane (C) and rhizosphere (D) samples.

(E, F) Principal coordinate analysis (PCoA) plots based on Bray-Curtis dissimilarity of KEGG Orthologs (KOs) for rhizoplane (E) and rhizosphere (F) samples.

**Supplement Figure 3. Abundance profiles of CAZymes associated with foxtail millet PGW.**
